# Micro-Evolution in Grasshoppers Mediated by Polymorphic Robertsonian Translocations

**DOI:** 10.1673/031.013.4301

**Published:** 2013-05-21

**Authors:** Pablo C. Colombo

**Affiliations:** Laboratorio de Genética, Departamento de Ecología, Genética y Evolución, Facultad de Ciencias Exactas y Naturales, Universidad de Buenos Aires, (1428) Ciudad Universitaria, Buenos Aires, Argentina

**Keywords:** chiasma frequency, environmental gradient, grasshoppers, microevolution, recombination, Robertsonian polymorphisms

## Abstract

This review focuses on grasshoppers that are polymorphic for Robertsonian translocations because in these organisms the clarity of meiotic figures allows the study of both chiasma distribution and the orientation of trivalents and multivalents in metaphase I. Only five species of such grasshoppers were found in the literature, and all of them were from the New World: *Oedaleonotus enigma* (Scudder) (Orthoptera: Acrididae), *Leptysma argentina* Bruner, *Dichroplus pratensis* Bruner, *Sinipta dalmani* Stål, and *Cornops aquaticum* Bruner. A general feature of these species (except *O. enigma*) is that fusion carriers suffer a marked reduction of proximal and interstitial (with respect to the centromere) chiasma frequency; this fact, along with the reduction in the number of linkage groups with the consequent loss of independent segregation, produces a marked decrease of recombination in fusion carriers. This reduction in recombination has led to the conclusion that Robertsonian polymorphic grasshopper species share some properties with inversion polymorphic species of *Drosophila,* such as the central-marginal pattern (marginal populations are monomorphic, central populations are highly polymorphic). This pattern might be present in *D. pratensis*, which is certainly the most complex Robertsonian polymorphism system in the present study. However, *L. argentina* and *C. aquaticum* do not display this pattern. This issue is open to further research. Since *C. aquaticum* is soon to be released in South Africa as a biological control, the latitudinal pattern found in South America may repeat there. This experiment's outcome is open and deserves to be followed.

## Introduction

The study of chromosomal polymorphisms occupies a distinguished place in the frame of the modern synthesis on evolutionary biology. Dobzhansky laid the empiric foundations of population genetics early in the twentieth century through the study of paracentric inversions of *Drosophila* (see review in Dobzhansky 1970). Heritable variability is the basis of population thinking, which debunked the pre-darwinian—and now obsolete— typological thinking. In the words of Mayr, “The populationist stresses the uniqueness of everything in the organic world” (Mayr 1970). For the population thinker, only the individuals composing populations have any reality. For the typologist, the type (eidos) is real and the variation an illusion, while for the populationist the type (average) is an abstraction and only the variation is real. Both visions are diametrically different (Mayr 1959).

Population thinking is the basis of the biological species concept, which regards species as a genetic pool, a reservoir of genes that can flow across generations due to sex and are subject to the Mendelian laws. Population studies are conducted within the frame of population genetics, the organizing principle of the modern synthesis (Dobzhansky 1970). The study of chromosomal polymorphisms and the assignation of different fitness values to different karyotypes was the pioneer manner in conducting genetic population studies. The emergence of molecular biology did not mean the demise of population cytogenetics; rather, cytogenetics grew as a complement to molecular biology. Molecular biology and classic cytogenetics united in what became known as molecular cytogenetics, a renewed way of studying chromosome polymorphisms with new tools available for the population cytogeneticist.

## Chromosome polymorphisms in insects

Most chromosome polymorphisms were studied in *Drosophila* species, given the ease of analyzing the polytenic chromosomes; however, the study of chromosome polymorphisms in short-horned grasshoppers (Orthoptera: Acrididae: Acridoidea) has been by no means negligible. Most chromosome polymorphisms in drosophilids are paracentric inversions (Powell 1997), while those of grasshoppers are mainly pericentric inversions and Robertsonian autosomal (Rb) translocations, and supernumerary heterochromatin (supernumerary segments and B chromosomes) (Hewitt 1979). This review focuses on Rb polymorphisms in grasshoppers. The clarity and crispness of meiotic configurations in Rb polymorphic grasshoppers has allowed the study of chiasma frequency and position in relation to the orientation of trivalents and multivalents in metaphase I (Hewitt 1979).

Roberts (1941) divided short-horned grasshoppers into two sections, Cryptosacci and Chasmosacci, on the basis of phallic structures. Both groups are karyologically conservative groups with respect to number and form. In fact, most male Cryptosacci have 23 chromosomes, and most female have 24, due to an X0/XX sex determination system (2n = 11 II + X0/XX). All chromosomes are acro- or telocentric. However, it should be noted that this conservatism is only superficial, given that DNA content and chromatin composition vary widely (John and Hewitt 1966). Upon this ancestral karyotype there may be derived karyotypes that vary in chromosome number due to fixed (i.e., non-polymorphic) chromosome rearrangements, most frequently Rb translocations (= centric fusions), where two acro- or telocentric non-homologous chromosomes fuse at the centromere level, usually with the elimination of a centric fragment (Hewitt 1979). The fusion of the X chromosome and an autosome is a frequent situation and creates a sexdetermination mechanism called “neoX-neoY” (See review in Castillo et al. 2010).

## The case of *Oedaleonotus enigma*

The first thorough study of Rb polymorphism from a population point of view was that of Hewitt and Schroeter (1968) in the grasshopper *Oedaleonotus enigma* Scudder (Melanoplinae: Acrididae). This species, confined to the western United States, bears a basic karyotype of 2n = 20 + neoX-neoY, and all chromosomes are telocentric with the exception of the neoX, which is sub-metacentric. A neoX-neoY bivalent regularly shows a heterochromatic X-arm and a euchromatic Y-arm. The corresponding homologue is neoY. The newly attached autosome, however, will remain euchromatic, as will its homologue, now the neo-Y. White (1954) and Sáez (1963) argued that the neoY becomes progressively more heterochromatic and genetically distinct due to the elimination of crossing over. The neoX-neoY bivalent of *O. enigma* remains euchromatic in its “new” portion, suggesting a recent origin of this sex determination system.

With respect to chromosome polymorphisms, the *O. enigma* population (Coalinga, Fresno County, California) had a polymorphism for a centric fusion between telocentrics 4 and 5 of the basic complement, for a centric shift in chromosome 8 (probably due to a pericentric inversion), for a supernumerary segment in the smallest member of the complement, chromosome 10, and for a B chromosome (Hewitt and Schroeter 1968). The behavior of the trivalent in the fusion heterozygote is quite regular, stemming from the fact that most of the orientation in the trivalent is alternate (i.e., disjunctional, the only orientation that may render viable gametes) and not linear (i.e., non-disjunctional, which would produce inviable gametes). The production of viable gametes in heterozygotes is necessary for the maintenance of any polymorphism, given that heterozygotes with reduced fitness would produce the loss of the polymorphism (Hedrick 1983, see further discussion below). Consequently, most metaphase II are euploid. Fusion heterozygotes are not, thus, subjected to negative heterosis, which is vital for the survival of the polymorphism. Remarkably, there is a relative excess of heterokaryotypes for the fusion when compared to the Hardy-Weinberg expectations (Hewitt and Schroeter 1968). An excess of heterozygotes might be indicative of heterosis, although not necessarily so; heterosis in turn would favor the maintenance of the polymorphism (Hedrick 1983).

Given that *O. enigma* normally has terminal chiasmata (see discussion below), no chiasma redistribution is needed. No interchromosomal chiasma effects were detected.

## The case of *Leptysma argentina* Bruner

*Leptysma argentina* Bruner (Orthoptera: Acrididae) is a semiaquatic acridid that lives and lays eggs in connection with Cyperaceae (it is a Leptysmine grasshopper, and thus its oviposition is endophytic). Its habitat extends from central Argentina and Uruguay to southern Brazil, and its habitat is semiaquatic, in connection with Cyperaceae (Amedegnato 1974; Roberts 1978). The karyotype of *L. argentina* is predominantly telocentric chromosomes (2n = 21 in males and 22 in females) with a unique sub-metacentric, probably a product of a centric fusion of two telocentrics (Bidau and Hasson 1984). Superimposed to this basic karyotype are three other polymorphisms in most studied populations: a centric fusion between chromosomes 3 and 6 of the basic complement (fusion 3/6), an interstitial supernumerary segment in the smallest chromosome of the complement (pair 11), and a medium-sized B chromosome (Bidau and Hasson 1984).

In respect to the effects of the polymorphic centric fusion on recombination, it was shown that it affects chiasma frequency and distribution. The carriers of this rearrangement display a lower chiasma count than the non-carriers, this effect being roughly additive, i.e., more marked in fusion homozygotes than in heterozygotes (Colombo 1989). The lower chiasma frequency is explained mainly by a decrease in proximal chiasma localization (Colombo 1990). Superimposed to this interchromosomal effect (i.e., not present in the chromosomes that are not involved in the centric fusion), a different intrachromosomal effect (on the chromosomes involved in the fusion) was found. Fusion homozygotes show a much lower proximal chiasma frequency in the submetacentric bivalent 3/6; furthermore, chiasma frequency decreases almost to 2 (one chiasma per submetacentric 3/6) in trivalent 33/6-6 of fusion heterozygotes (Colombo 1987, 1990). As a consequence of this decrease, average chiasma frequency and position decrease along with the increase of fusion 3/6 frequency in different populations (Colombo 1989).

This double decrease of proximal chiasma frequency would lead to a diminution of genetic recombination in fusion carriers. This effect should be added to an automatic consequence of a centric fusion—the reduction in the number of linkage groups. In fact, a centric fusion causes two originally unlinked chromosome pairs that would otherwise segregate independently to become physically linked, which would reduce drastically the number of chromosome combinations in the gametes. This reduction is intensified because proximal chiasmata in the trivalent (the only case in which genetic recombination between the fused metacentric and the un-fused acrocentric might arise) are almost suppressed. Hence, polymorphic Rb translocations would have a similar effect as polymorphic inversions, namely a suppression of recombination between the two pairs of chromosomes involved in the rearrangement. The arisal of coadapted gene complexes might provide a possible explanation of why Rb polymorphisms occur in nature (see discussion below) (Dobzhansky 1970; Colombo 1989; Bidau 1993).

## Correlation with morphological changes

All the focus on chiasma effects is due to the fact that in grasshoppers, as in other organisms that are not amenable to genetic analysis, chiasma frequency is the only measurable variable to assess the amount of genetic recombination. This measuring is possible because meiotic figures are extremely clear in grasshoppers and chiasma distribution can be detected with accuracy (Hewitt 1979).

Another reason for the emphasis on chiasma effects is that, until recently, it was generally accepted that chromosomal rearrangements had no effect on the external phenotype (Lande 1979). However, in 1989, it was detected that fusion 3/6 in *L. argentina* was clear and consistently related to changes in body size (Colombo 1989). Centric fusion 3/6 is associated with an increase in body size in carriers, and this effect is additive, i.e., its effect is more marked in fusion homozygotes than in heterozygotes in both males (Colombo 1989, 1997) and females (Colombo et al. 2004). This relation is the reason why body size is correlated with fusion 3/6 frequency in some populations (Colombo 1989, 1997).

In organisms such as *Drosophila* that are better suited to genetic research than grasshoppers due to their life-history characteristics, the effects of paracentric inversions on selection components, such as longevity, fertility, larval viability, hatching, and so on, have been thoroughly studied (see Powell 1997 for a review). Temperate-region grasshoppers, such as *L. argentina,* can also be studied because the grasshopper's generations are discrete and synchronized. By studying fusion 3/6 frequency in young and aged males in two populations of this species, male longevity was assessed, determining that fusion 3/6 increases male longevity in carriers (Colombo 1993, 2002). A later study identified the effect of fusion 3/6 on body size-related traits, such as femur length, as the cause for this increase in longevity of fusion carriers (Norry and Colombo 1999). Body size-related traits also seem to be the reason for the effect of fusion 3/6 on other selection components. Mating success in *L.* argentina was increased in males (Colombo et al. 2001) and females (Colombo et al. 2004) correlated with an increase in femur length associated to this fusion.

## The case of *Dichroplus pratensis*

Although the karyotype of *Dichroplus pratensis* Bruner (Orthoptera: Acrididae) was described by Mesa (1956) and Sáez (1956), and a hint of its complex system of Rb polymorphisms and polytypisms was summarily exposed by Sáez and Pérez Mosquera (1970), it was not until 1988 (Bidau and Mirol 1988) that many papers started to emerge describing the unusual characteristics of *D. pratensis.*

The male standard karyotype of *D. pratensis* consists of 2n = 18 + X0 telocentric chromosomes, divided into 6 large (L1- L_6_) and 3 small (S7- S9) chromosome pairs; the X is slightly smaller than L_4_ (Bidau and Martí 1995; Martí and Bidau 1995). L bivalents have a proximal distal chiasma pattern, while S bivalents always show a single chiasma.

So far, 63 natural populations from different habitats and latitudes have been chromosomally examined (Martí and Bidau 2001a, 2001b; Bidau and Martí 2002), and eight Rb translocations were identified among all 6 long autosomes (1/2, 1/4, 1/6, 2/4, 2/5, 3/4, 3/5, and 5/6). There may be up to 4 different combinations in one population, which creates monobrachial homologies, i.e., pairs of metacentrics that are homologous for only one arm, for example fusions 5/6 and 1/6, that are homologous for only arm 6. Monobrachial homology arises in hybrid zones. With few exceptions (see below), all populations are usually polymorphic for one to four fusions whose characteristics are very different. The frequency of fusion chromosomes is generally high, except in marginal populations (see below), where most or all individuals may bear an all-telocentric karyotype (Martí and Bidau, 2001a, 2001b; Bidau and Martí 2002). Some fusions are widely distributed (3/4, 1/6), while others are restricted to small isolated areas (1/2) or even to a single population (3/5). No evident geographic pattern has been detected, although fusions 1/6 and 3/4 are typical of the (temperate) central area of the species' distribution, while 5/6 is only found in the southernmost Patagonian populations.

It was suggested that the fusions of *D. pratensis* follow a strict central-marginal model (see below). The largest wealth of fusions per population, as well as their highest frequencies, is in central-eastern Argentina (ecologically more favorable for the species), whereas northwards, westwards and southwards, the frequency and diversity of rearrangements decrease clinally. At the extreme margins of the geographic range, fusions practically disappear, marginal populations being monomorphic and all-telocentric. Furthermore, average fusion number per individual and average chiasma frequency per cell (a measure of recombination; see below) are negatively correlated (Bidau et al. 2003). Hence, marginal populations have a higher level of recombination, which is probably reflected in the degree of morphological variability within populations (Martí and Bidau 2001a, 2001b; Bidau and Martí 2002, 2005).

## Effects of Rb translocations on chiasma frequency and distribution

Since the behavior of heterozygous Rb configurations is strongly affected by chiasmata, the effects of the fusions on chiasma frequency and localization were studied. The results may be summarized as the following:

1) Telocentric bivalents have a predominant proximal-distal chiasma distribution (Bidau 1990, 1993; Bidau and Martí 1995, 2002; Martí and Bidau 1995, 2001a, 2001b).
2) Fused bivalents and trivalents have less total and proximal chiasmata than telocentric ones, and show a shift of chiasmata towards distal positions in both sexes. No significant differences between fusion bivalents and trivalents exist, nor between sexes.
3) The intrachromosomal reduction in total chiasma frequency depends on the size of the involved telocentrics, as telocentric length and chiasma frequency per bivalent or trivalent are significantly correlated.
4) Fusions have a homogenizing effect, producing the same chiasma frequency in all chromosomes and chromosome combinations (about one distal chiasmata per chromosome arm) in both sexes (Bidau 1990; Bidau and Martí 1995, 2002; Martí and Bidau 1995).
5) There are no differences between males and females with identical Rb karyotypes, with the exception of the X bivalents of females, which has a typical proximal-distal chiasma pattern.
6) Intrachromosomal recombination within populations, when assessed by a chiasma position-based recombination index, is negatively correlated with the number and frequency of fusions (see below). This effect is added to the instant reduction of intrachromosomal recombination produced by the combination of two linkage groups in one due to each individual centric fusion.

## The hybrid zones: chromosomal and genetic studies

An extensive and complex Rb polymorphism involving fusions 1/2, 3/4, 5/6 and 1/6 that occurs in Sierra de la Ventana (Buenos Aires province, Argentina) has been interpreted as the result of hybridization between two chromosomal races sharing fused chromosomes with monobrachial homologies. In this region, a southern race, geographically very restricted between Sierra de la Ventana and the sea, and polymorphic for fusions 1/2, 3/4 and 5/6, contacts the widespread northern race polymorphic for fusions 1/6 and 3/4. Thus, because of reproductive interactions between individuals of both races, complex Rb heterozygotes with reduced fertility due to the formation of multiples are produced in the hybrid zone (Bidau 1991, 1996).

Within the hybrid zone, chromosome frequencies vary widely over relatively short distances and altitudes, with fusions 1/2 and 5/6 generally associated with higher altitudes (rocky tops of hills) (Tosto and Bidau 1991). The irregular distribution of both races within the hybrid zone possibly is because of the complexity and heterogeneity of the Sierra de la Ventana environment, which transitions between radically different biogeographic zones (the grasslands of the Pampas to the north and the Patagonian steppes to the south). Hence, the hybrid zone has been interpreted as a mottled zone (Mirol and Searle 1995) with very complex relationships between both races, whose internal distribution reveals a notorious spatial heterogeneity (Tosto and Bidau 1991).

Many populations include individuals from both races, as well as complex structural chromosomal hybrids. The latter are of the following three types, according to the involved monobrachial homologies (Bidau et al. 2003):

1. Type “561”; besides the chromosomes shared by both races, they have two Rb metacentrics (1/6 and 5/6) and two telocentrics (1 and 5) that engage in the formation of a quadrivalent (5-5/6-6/1-1).
2. Type “612”; quadrivalent: 6-6/1-1/2-2.
3. Type “5612”; quinquivalent: 5-5/6-6/1-1/2-2.

The frequency of non-alternate (linear) orientations in metaphase I is high for allmultivalent types (Bidau 1991, 1996), especially for 612 and 5612. Desynapsis is very frequent (up to 18% of the cells in some individuals) and it always involves the telocentric chromosomes, particularly 5 and 6. Linear orientations of multivalents in prometaphase I are even more frequent than in metaphase I in all hybrids, reaching frequencies of 75% in some specimens (Bidau 1991, 1996; Martí and Bidau 1995).

Abnormal (aneuploid and diploid) secondary spermatocytes are significantly more abundant in complex hybrids than in regular heterozygotes, and a highly significant positive correlation exists between their frequency and the frequency of abnormal (non-disjunctional) first metaphases. Nevertheless, the frequency of abnormal spermatocytes II is almost always lower than that of linear orientations, suggesting again that further reorientation occurs at metaphase I.

## Genetic studies in the hybrid zone

The genetic structure of populations of *D. pratensis* from northern and southern races in the hybrid zone was examined (Chiappero et al. 2004; Miño et al. 2011). Genetic differentiation among parental races was significant, even though no fixed alleles for any particular race were found. Hybrid populations are genetically more similar to the southern than to the northern race. This difference is most likely because the northern race probably colonized the areas previously occupied by the southern. In this scenario, the populations from within the hybrid zone have been in contact with the northern race for longer. The results support the hypothesis that the northern race invaded its present range, displacing the southern one that at present is only represented within the zone by relictualpopulations on the top of the hills (Bidau et al. 2003).

## Meiotic behavior of single and multiple Rb heterozygotes

The orientation at metaphase I of six heterozygous fusions in males and two in females of *D. pratensis* was studied. Linear (non-convergent) orientation at prometaphase I varied between 4% and 40% for any individual trivalent in different individuals, and the mean values for four different trivalents were not statistically significantly different. Reorientation takes places at two points, in metaphase I and in second spermatocytes. In all cases, metaphase I linear orientations are significantly lower than in prometaphase I; furthermore, the frequency of aneuploid and diploid secondary spermatocytes are always lower than that expected from the metaphase I orientations (Bidau and Mirol 1988; Bidau and Martí 1995). This discrepancy between the metaphase I and metaphase II data suggests that additional reorientation must occur during metaphase I, and that the effects on fertility of a given rearrangement must not be inferred exclusively from its metaphase I behavior (Bidau and Martí 1995).

In double and triple heterozygotes, the situation is similar. As expected, the frequencies of linear orientation are higher than those of single heterozygotes and roughly additive for all combinations of fusions analyzed. The levels of metaphase I linear orientation can be as high as 36% in some individuals (Bidau and Mirol 1988; Mirol and Bidau 1994; Bidau and Martí 1995). Also, in these individuals, a considerable degree of metaphasic reorientation seems to take place, and the frequencies of anomalous spermatocytes II are relatively low, although higher than those observed in single heterozygotes (Bidau et al. 2003).

## Factors affecting meiotic orientation of Rb multivalents

Orientation of Rb multivalents is conditioned by the distance between centromeres, which in turn depends on the size of the involved chromosomes and the localization and frequency of chiasmata, which determine the symmetry of the configuration (Narasinga Rao and Sybenga 1984; Arundhati et al. 1986; Bidau 1991, 1996; Mirol and Bidau 1991; Bidau and Martí 1995).

The number of centromeres in the multiple is also relevant. Multiple regression analysis involving the comparison of linear orientations in four trivalent types (1/2, 3/4, 5/6, and 1/6) at prometaphase and metaphase I in relation with arm length, arm ratio, and frequency of proximal chiasmata, revealed that the sole factor significantly affecting orientation in both stages was proximal chiasma frequency (Mirol and Bidau 1992).

Higher order Rb multivalents also show a strong dependence on proximal and interstitial chiasmata for their orientation and, in this case, a highly significant positive correlation for all multivalent types occurs. Total length of the configuration is also important because linear orientation frequencies increase with multivalent size (561 < 612 < 5612; see below) (Bidau 1991, 1996; Bidau and Martí 1995).

## The case of *Sinipta dalmani* Stål

In *Sinipta dalmani* Stål (Acrididae: Gomphocerinae), which is widely distributed throughout Argentina and Uruguay, a polymorphism for a centric fusion between the X chromosome and an autosome was found in three populations confined to a restricted protected area in central east Argentina (Remis 1990, 2008). The centric fusion involving the sex chromosome is present in a polymorphic state (Remis 1990), a unique situation in Orthoptera, although there are several instances in which fixed X-autosome fusions allow the distinction between related Orthoptera races or species (Hewitt 1979). Especially remarkable is the case of *Podisma pedestris,* a flightless alpine grasshopper with two chromosomal races, one bearing an X0 and the other a neo-XY sex chromosome system (John and Hewitt 1970; Hewitt 1975; Mason et al. 1995); however, this is not a polymorphism but a polytypism. A similar case has recently been described in the brachypterous grasshopper *Podisma sapporensis* (Kowalczyk et al. 2008; Warchalowska-Sliwa et al. 2008b). The frequency of the fusion in the different samples of *S. dalmani* was low, and there were no statistical differences among populations or among years. This fusion was associated both with an increase in terminal chiasma frequency in the fused pair (Remis 2008) and with a decrease in the frequency of functional sperm (Remis 1993). Thus, based on the temporal stability, the influence on sperm formation, and the particular effect on genetic recombination, it is tempting to suggest a non-neutral explanation for the maintenance of this fusion at a low frequency (Remis 1993).

## The case of *Cornops aquaticum* Bruner

The water-hyacinth grasshopper, *Cornops aquaticum* Bruner (Orthoptera: Acrididae), is another New World leptysmine grasshopper that lives, feeds, and oviposits on plants of the genus *Eichhornia* (Adis and Junk 2003; Adis et al. 2004). *C. aquaticum* has been poised for release in Africa as a natural control of waterhyacinths (Oberholzer and Hill 2001), which have become a serious water weed (Centre et al. 2002). In its natural environment, *C. aquaticum* lives between 23° N and 35° S, i.e., between the south of Mexico and east-central Argentina and Uruguay. This case is the most recently studied case of Rb polymorphisms in grasshoppers (Colombo 2007, 2008, 2009), although the occurrence of polymorphic Rb rearrangements in a Uruguayan population of this species was first reported by Mesa (1956) and Mesa et al. (1982). In the southernmost extreme of its geographic distribution, three polymorphic Rb rearrangements were found that follow a north-south cline (Colombo 2008). These centric fusions severely affect chiasma distribution in the chromosomes affected by the rearrangements, shifting chiasmata from proximal and interstitial to more distal positions (Colombo 2007).

*C. aquaticum* has 2n = 23 chromosomes in males and 24 in females, with a X0/XX sex determination system. All chromosomes are acro-telocentric. Upon this basic karyotype, three Rb translocations took place between pairs 1/6, 2/5, and 3/4. (Colombo 2008). These polymorphisms are restricted to the lower course of the Paraná River, between Rosario and Buenos Aires. Fusion frequencies increase southwards, thus showing a geographical cline. The polymorphisms were mostly in keeping with Hardy-Weinberg and gametic phase equilibria (Colombo 2008). The rearrangements cause a drastic chiasma re-patterning in the fusion bivalents (or trivalente), reducing proximal chiasma frequency (Colombo 2007). Recombination is also reduced due to the loss of independent segregation. A recombination index (Colombo 1992) that takes into account both factors correlates negatively with the number of pairs affected by fusions among populations (Colombo 2008).

## Chiasma studies in C. *aquaticum*

Chiasma reorganization in trivalents of heterozygotes would be necessary if the polymorphisms were to be maintained, since proximal chiasmata would lead to non-disjunctional orientation of the trivalent. Chiasma frequency and distribution were analyzed in five Argentine populations. This study revealed a strong redistribution of chiasmata in fusion carriers, with a reduction of proximal chiasma frequency and an increase of distal ones in the fusion bivalents and trivalents, when all three karyotypes were compared. However, when only fusion bivalents and trivalents were compared, proximal chiasma frequency reduction in trivalents of heterozygotes is more marked than in the submetacentric bivalents of fusion homozygotes (Colombo 2007). It is argued that proximal chiasma frequency reduction (with respect to unfused bivalents) in fusion bivalents may be due to interference across the centromere (Colombo and Jones 1997; Colombo 2007), but this may only be the case in fusion homozygotes, as interference need a complete synaptonemal complex in order to operate. Proximal chiasma reduction in heterozygotes is due to another cause, namely an adaptation to the polymorphic situation (Colombo 1993a), or else to synaptic problems in fusion bivalents and trivalents, as proposed in the case of *D. pratensis* (Martí and Bidau 2001c).

## Study of trivalent orientation in metaphase I in *C*. *aquaticum*

Twenty-one out of the 27 males used in this study were captured from two highly polymorphic populations of *C. aquaticum.* Three other males from a mostly monomorphic population (all-metacentric) of *C. aquaticum* were used in order to have some representatives of tives of the “zero trivalent” class. In each of the males, we sought to find out whether there was any relationship between the frequency of linear orientation and the number and position of chiasmata. It was clear from multivariate and univariate analyses that a high chiasma number favors linear orientation (as shown by standard correlation with arcsin transformation due to the presence of proportions); among these chiasmata, proximals and interstitials are the ones that would lead to linear orientation, distal chiasmata being associated with convergent orientation.

Individuals with no heterozygous fusions, and with one, two, and three trivalents, were compared with respect to their formation of diploid or tetraploid spermatids. The regression of the proportion of abnormal spermatids on the number of trivalents was significant, as expected. There seems to be no interaction between trivalents; rather, the effects are roughly accumulative. The regression of the percentage of abnormal spermatids on the percentage of linear orientation was not significant (Colombo 2009).

Aneuploidy was rare in metaphase II and anaphase I in *C.* aquaticum, suggesting that segregation of trivalents is generally normal. A total of 149 metaphase II plates were observed, and aneuploidy was seen in only five cases. Anaphase I cells were rare, but their quality was much better. A total of 53 anaphase I cells were observed. Segregation of trivalents was always normal, showing the submetacentric pointing towards one pole and the two acrocentrics migrating towards the other. Hence, as in *D. pratensis,* a great deal of reorientation of linearly oriented trivalents must be taking place. All the results from this and other studies are consistent in suggesting that the polymorphic centric fusions of *C. aquaticum* are indeed stable ones (Colombo 2009).

## Comparative study of spontaneous and polymorphic centric fusions

Polymorphic centric fusions usually produce a marked chiasma redistribution from proximal and interstitial to more distal positions with respect to the centromere in the fusion trivalent of heterozygotes (see below). However, there is evidence that this redistribution is not due to an automatic effect of the rearrangement on chiasma position. Southern (1967) observed no intrachromosome effect of the spontaneous M5/M6 fusion on the chiasma pattern in *Myrmeleotettix maculates.* Polani (1972) also failed to find chiasma restriction in a heterozygote for a newly arisen 6–15 centric fusion in *Mus musculus.* Futhermore, it does not seem to be true for a 2/3 heterozygote of the grasshopper *Staurorhectus longicornis* (Vilardi 1984). Peters (1982) reported the recurrence of centric fusions in interpopulational synthetic hybrids of the pyrgomorphid *Atractomorpha similis.* Although there are no details about presumed effects on chiasma distribution, it is inferred from the text and the figures of Peters (1982) that the high frequency of proximal and interstitial chiasmata remains unchanged. A spontaneous centric fusion in the grasshopper *Valanga nigrocornis* had a strong interchromosome effect, reducing chiasma frequency of both long and medium sized chromosomes, but no intrachromosome effect was reported (Teoh and Yong 1983). Finally, López Fernández et al. (1984) found a slight reduction in proximal chiasma frequency in the short arm of the metacentric originated through a spontaneous centric fusion between pairs 5 and 8 of the grasshopper *Chorthippus jucundus.* Additionally, a few cases of spontaneous X-autosome fusions were observed. John and Hewitt (1968) reported a case in *Arcyptera [Pararcyptera] kheili.* No shift of chiasmata to distal positions was observed. The same seemed to occur in a possible spontaneous mutant studied by Mesa and Mesa (1967) in *Leiotettix politus.* In *Baeacris punctulatus,* the mutant showed strict distal localization of the single chiasma, but this result could be due to a general tendency for distal chiasma formation in the species (Castillo et al. 2010).

In two cases, a spontaneous and a polymorphic centric fusion were tested for chiasma effects in the same species: one male in a population of *L. argentina* heterozygous both for a 5/7 spontaneous fusion and the 3/6 polymorphic one (Colombo 1987). The orientation of the 5–5/7–7 trivalent in metaphase I was irregular (36% linear orientation) and no intrachromosomal effect on chiasma frequency or distribution was detected. In contrast, trivalent 3–3/6–6 orientation in the double heterozygote was much more regular (3%), not significantly different from non mutant heterozygotes. As already reported, there is a marked effect of the polymorphic fusion reducing the frequency of chiasmata in the trivalent and shifting them to more distal positions (Colombo 1987).

In *Sinipta dalmani,* a heterozygous individual was found for a spontaneous M6/M7 centric fusion. In the same and nearby populations, another polymorphic X/M5 centric fusion took place (Remis 1990). Hence the mutant and the polymorphic centric fusions occur in the same population, but not in the same individual. Again, the spontaneous M6-M6/M7-M7 trivalent shows an irregular meiotic behavior (77% of linear orientation) leading to 51% of aneuploid metaphase II (in the case of *L. argentina,* MII plates are usually not analyzable), and chiasma frequency and position remain unaffected. By contrast, the polymorphic X/M5 fusion triggers a significant reduction in the frequency of chiasmata and their displacement to terminal position (Remis 1990, 2008).

Thus, in both cases, a chiasma redistribution accompanied a stable (convergent) behavior of the polymorphic trivalent, as already shown in *D. pratensis* and *C. aquaticum.* However, no change in chiasma frequency or position was noticed in the spontaneous centric fusions trivalents, accompanied by a high incidence of linear (non-linear, non-convergent) orientation. These findings suggest that the chiasma effects found in both cases are due to an adaptation to the polymorphic situation (see below).

## Chiasma redistribution due to Rb polymorphisms

Rb translocations are frequent among the rearrangements that are involved in the evolutionary divergence throughout the animal and plant kingdoms (Capanna 1982; Baker and Bickham 1986; King 1993). When hybrids between species or chromosomal races that differ in one or several Rb rearrangements arise, the behavior of the resulting trivalent(s) is frequently erratic, leading to imbalanced gametes due to linear configurations in metaphase I. Therefore, fusion heterozygotes should have negative heterosis for fitness. Given that polymorphisms with negative heterosis are unstable (Hedrick 1983), there should hardly be polymorphic centric fusions; however, this is not the case (Hewitt and Schroeter 1968; Mayr et al. 1984; Colombo 1989, 2007; Bidau 1990; Remis 1990; Fan and Fox 1991; Nachman 1992; Pascoe et al. 1996; Narain and Fredga 1998, to mention only a few). Evidently, in these cases, the depressing effects of structural heterozygosis on fitness are somehow suppressed. In fact, when trivalent orientation is studied, convergent orientation is the norm and linear orientation the exception (Bidau and Mirol 1988; Mirol and Bidau 1991, 1992; Colombo 2009).

This result is partly because convergent orientation requires distal chiasmata, given that too many proximal chiasmata (with respect to the centromere) would spatially obstruct the bending of the trivalent at the level of the centromere, which leads to balanced segregation. Frequently, centric fusions trigger a chiasma redistribution from proximal to more distal positions both in grasshoppers (Hewitt and Schroeter 1968; Bidau 1990; Colombo 1989, 1990, 1993, 2007) and in mice (Davison and Akeson 1993; Dumas and Britton-Davidian 2002). What follows is a brief discussion about the possible causes of this redistribution.

## Chiasma redistribution in centric fusion carriers: a common feature

Among polymorphic centric fusions, the frequency of chiasmata (especially proximal) is always reduced (Hewitt and Schroeter 1968; Colombo 1989, 1990, 1993, 2007; Bidau 1990; Bidau and Martí 2005). This reduction was suggested to be due to an adaptation to the polymorphism (Colombo 1989, 1993). Alternatively, it was attributed to a direct effect of delayed synapsis in the trivalents (Davisson and Akeson 1993; Bidau and Martí 2001). However, this feature is restricted to Rb polymorphisms.

The populations of the European house mouse (*Mus domesticus*) are polymorphic and/ or polytypic for several Rb rearrangements (Garagna et al. 2001). Studies on this species show that both chiasma conditions and genetic recombination are affected by Rb rearrangements, leading to a decrease in the number of proximal chiasmata (Davisson and Akeson 1993; Bidau et al. 2001; Dumas and Britton-Davidian 2002; Bidau and Martí 2005). However, they frequently deal only with fusion and acrocentric homozygotes by comparing populations that differ in several Rb rearrangements. Instead, when hybrids between Rb races of mice are compared, proximal chiasma frequency increases rather than decrease in the trivalents of heterozygotes (Bidau et al. 2001). Furthermore, it has been claimed, at least in some cases, that a partial asynapsis, or rather a delay in pairing due to structural heterozygosis, is correlated with proximal chiasma suppression (Davisson and Akeson 1993; Bidau and Martí 2001). But is asynapsis a cause of the proximal chiasma suppression, or a consequence? In species with strict chiasma localization, it is usually accompanied by asynapsis, though in these cases no structural hybridity obstructs synapsis (Fletcher 1977; Del Cerro et al. 1997; Viera et al, 2009). We predict that, when proximal chiasma suppression in Rb trivalents is observed, it will be in heterozygotes of Rb polymorphisms, rather than in hybrids (natural or otherwise) between different chromosomal races. This prediction can be easily falsified by testing chiasma distribution in hybrid mice. So far, the only such studies revealed that chiasma frequency per chromosome arm in trivalents is the same or even higher than that of telocentrics, whereas metacentric bivalents have a lower chiasma frequency per chromosome arm (Wallace et al. 1992, 2002; Bidau et al. 2001; Castiglia and Capanna 2001), thus confirming our hypothesis.

However, a general feature of the meiosis of polytypic or polymorphic Rb heterozygotes is that fusion bivalents also show a more distal redistribution of chiasmata, although the change is not necessary for the maintenance ofthe polymorphism. This change is milder than that observed in trivalents (Bidau 1990; Colombo 1993, 2009; Bidau et al. 2001, Bidau and Martí 2005) and is also present in fusion homozygotes even though the trivalents of heterozygotes do not show proximal chiasma suppression (Bidau et al. 2001). To explain this fact, we take into account that chiasma interference can act through the centromere (Colombo and Jones 1997), contrary to the classical belief, which can be traced back to Mather (1938). Broman and Weber's (2000) study on human meiosis using genetic markers confirmed that interference was “blind” to centromeres. Hence, we propose that the chiasma redistribution found in Rb bivalents (when compared to the homologous unfused chromosomes) is due to the action of interference across the centromere. This proposal cannot be the case in trivalents given that interference and a complete synaptonemal complex are usually connected (Sym and Roeder 1994; Börner et al. 2004). Hence, we also predict that, when chiasma redistribution is seen in Rb bivalents and trivalents, it is of a diverse nature because they are due to different causes.

## The central-marginal model in Robertsonian polymorphic grasshoppers

In natural populations of *Drosophila,* it has been found that the most central populations (both from an ecological and a geographical point of view) tend to be more polymorphic for paracentric inversions, whereas the marginal populations tend to monomorphism. This was termed the “central-marginal model” (Powell 1997). Da Cunha and Dobzhansky (1954) proposed, in *Drosophila willistoni,* that the level of inversion polymorphism is directly related to the diversity of the habitat occupied by the populations. The idea is thatthe greater the environmental diversity, the more inversions it can maintain due to diversifying selection. Another explanation of the central-marginal pattern, by Carson (1955, 1959), states that central populations are well adapted and hence recombination disrupts well-adapted combinations of genes. As inversions suppress recombination in the mutually inverted sequences of heterozygotes, the low frequency of inversions in marginal populations would be reflecting the need for new combinations of genes in populations that are challenged by environmental instability.

Centric fusions also reduce recombination due to the lack of independent segregation of two pairs of chromosomes that before the rearrangement used to migrate independently. Furthermore, as has been sufficiently exposed, polymorphisms for centric fusions reduce proximal and interstitial chiasma frequency in the trivalent; contrary to distal chiasmata, proximal and interstitial are the only ones that may produce genetic recombination. Consequently, they are expected to produce the same effects as polymorphic inversions, allowing the preservation of co-adapted gene complexes (i.e., groups of genes that work together interrelated by tightly woven threads of epistasis, thus allowing the adaptation to different environments, and that occasionally are tightly linked and inherited together, thus forming supergenes; Dobzhansky 1970), and thus amenable to display a central-marginal pattern.

However, in *L. argentina,* it was found that the southernmost populations had a higher fusion frequency and the lowest degree of recombination (Colombo 1989), even if in many aspects it could be said that these southern populations from temperate climates are marginal for a species whose distribution is mainly tropical and subtropical.

However, a different case seems to be that of *D. pratensis.* This species is polymorphic and/or polytypic for eight different Rb rearrangements throughout Argentina, and all of them cause a chiasma re-patterning leading to a reduction in recombination (Bidau 1990). Cytogeographic study (Bidau et al. 1991; Bidau and Martí 2002) found that heterozygosis in central populations is highest, diminishing in clearly marginal ones, such as those sited in northern Argentina and in Patagonia; this geographic pattern was interpreted by the authors as a central-marginal one. They analyzed chiasma frequency (dependent on the frequency of centric fusions) in relation to the variation of six exomorphological characters (expressed as their Coefficients of Variation). Central populations, which inhabit ecologically optimal regions, exhibit a high level of fusions and consequently low chiasma frequency. On the other hand, marginal populations from Patagonia and the Precordillera have very low levels of chromosomal polymorphism and hence high chiasma frequencies (Xt = 11.66 in the southernmost population at 45° 57′ S, and X t= 12.01 in the northernmost one sited at 23° 55′ S). The progressive increase in recombination frequencies toward the margins of the geographic range is negatively correlated with the decrease in frequency of chromosomal polymorphisms, but also positively correlated with augmented levels of morphological variability. This evidence would indicate a central-marginal pattern.

In *C. aquaticum* this central-marginal pattern does not seem to be the case, and in many aspects *C. aquaticum* resembles *L. argentina.* It is clear that the mouth of the Paraná river is a marginal environment for this species—in fact, it is by far the southernmost extreme of its distribution—and yet it is there where the highest frequency of centric fusions was found. Furthermore, populations from the center of the geographical distribution, such as those from the state of Pernambuco in Brazil, at latitude 8° S (Rocha et al. 2004) and Trinidad and Tobago at latitude 10.30° N (Colombo 2008), do not show any chromosomal polymorphism. In *C. aquaticum,* recombination is lowest in a place where marginality is more extreme (Buenos Aires, latitude 34° S), so it clearly contradicts the central-marginal model (Colombo 2008). Contrary to *D.* pratensis, which seems to be a fairly settled case, the meaning of this cline and that of *L. argentina* awaits for further sampling and research (see Colombo 2012 for further discussion of this issue).

In species of *Drosophila* where a clinal variation for inversions was found, notably in *D. subobscura* (Prevosti et al. 1988), the clines have tended to reproduce wherever this species was transported accidentally by human transport. In fact, a north-south cline for paracentric inversions in Europe has repeated itself in the Pacific coast of North America, and it reversed and became a south-north cline in the Pacific coast of South America. This invasion of the New World by an Old World species has been called by Ayala et al. (1989) “a grand experiment in evolution.” A similar pattern of latitudinal clines in both hemispheres exists for *D. melanogaster* (Lemeunier and Aulard 1992). As *C. aquaticum* is poised for introduction in South Africa as a biological pest control (Oberholzer and Hill 2001), it is tempting to say that the clines found in South America may repeat there, if only the founder populations contained the Rb polymorphisms. We will be looking forward to the outcome of this new experiment in evolution.
